# The Role of Interleukin 6 (IL6), Cancer Antigen—125 (CA-125), and Human Epididymis Protein 4 (HE4) to predict tumor resectability in the advanced epithelial ovarian cancer patients

**DOI:** 10.1371/journal.pone.0292282

**Published:** 2023-10-04

**Authors:** Syamel Muhammad, Reyhan Julio Azwan, Rauza Sukma Rita, Restu Susanti, Yusrawati

**Affiliations:** 1 Obstetrics and Gynecology Department, Medical Faculty of Andalas University, Padang, West Sumatera, Indonesia; 2 Biomedical Science Department, Medical Faculty of Andalas University, Padang, West Sumatera, Indonesia; 3 Nephrology Department, Medical Faculty of Andalas University, Padang, West Sumatera, Indonesia; 4 Fetomaternal Division, Obstetrics and Gynecology Department, Medical Faculty of Andalas University, Padang, West Sumatera, Indonesia; Sapienza University of Rome: Universita degli Studi di Roma La Sapienza, ITALY

## Abstract

**Introduction:**

A study of tumor resectability in pre-operative patients with advanced epithelial ovarian cancer is required to predict primary surgical benefits accurately. This study aims to investigate IL6, CA-125 and HE4 to predict tumor resectability in the pre-operative patients with advanced epithelial ovarian cancer.

**Methods:**

This cross-sectional study was conducted in the polyclinic, oncology and gynecology inpatient room of Dr. M. Jamil Padang Hospital from June until December 2022. Advanced epithelial ovarian cancer stage based on histology result from FIGO stages IIIB–IVA. IL6, CA-125, and HE4 were measured using ECLIA (electrochemiluminescence immunoassay). Categorical data were assessed using Chi-square and Mann-Whitney tests. Numerical variable correlations were analyzed using Pearson Correlation tests. While the correlation between numerical and nominal variables was analyzed using the Eta correlation test. A p-value of <0,05 was considered a significant correlation. The cut-off value of serum IL6, CA-125, and HE4 was determined with a ROC curve. The sensitivity and specificity of each clinical parameter were calculated.

**Results:**

There was a significant difference in IL-6 (1328 vs 752 pg/ml; p<0,001), CA-125 (1260,5 vs 819,5 U/ml; p<0,001), and HE4 levels (1320 vs 760 pmol/L; p<0,001) between patients with tumor resectability of > 1 cm (suboptimal) vs < 1 cm (optimal). There was a correlation between IL6 (r = 0,832), CA-125 (r = 0,716), and HE4 (r = 0,716) with tumor resectability.

**Conclusion:**

Measuring IL6, CA-125, and HE4 levels is useful for clinicians to predict tumor resectability in pre-operative patients with advanced epithelial ovarian cancer.

## Introduction

Epithelial ovarian cancer is a neoplasm that develops in the epithelial tissue covering the outside of an ovary. It is the most common type of ovarian cancer, contributing to 90% of ovarian cancer types [1]. Worldwide, ovarian cancer was the third most common gynecological cancer in 2020, with 313,959 new cases of ovarian cancer recorded globally [2]. In the United States, approximately 22,240 new cases of ovarian cancer were diagnosed and 14,070 ovarian cancer deaths [1]. In Asia/Pacific, the cases of ovarian cancer were 9.2/100,000, and in Indonesia, the incidence and mortality are the third-highest, accounting for 7% of malignancy in women, with 9581/14,896 deaths in 2020 [3].

Tumor markers, substances produced by cancer cells or normal cells in response to cancer growth, play a crucial role in diagnosing and managing epithelial ovarian cancer. Their advantages include early detection, monitoring treatment response, recurrence surveillance, non-invasive testing, and providing prognostic information. However, these markers also have limitations, such as lack of specificity, potential for false results, variability among individuals, limited effectiveness in early-stage detection, and cost and availability concerns. To maximize their benefits, tumor markers should be used in conjunction with other diagnostic methods, ensuring accurate and comprehensive care for ovarian cancer patients [4, 5].

A standard diagnostic assessment for a woman with suspected advanced ovarian cancer includes a physical examination, ultrasonography, serum cancer antigen (CA-125) measurement and imaging analysis (CT scan or MRI). To obtain an accurate prediction of primary surgical benefits, these standard procedures had low accuracy in predicting the outcomes of primary surgery with a deposition of tumor resectability > 1 cm. Furthermore, the location of the tumor affects the metastatic tumor resectability, and an optimum cytoreduction is challenging with a spreading disease into the diaphragm, liver parenchyma, small intestinal surface area, lesser omentum, or hepatic portal [6].

Cancer antigen 125 (CA-125) is a protein detected in the blood and commonly used to detect early ovarian cancer. The elevation of CA-125 is also associated with other malignancies, such as pancreatic, lung, breast, colorectal, and benign ovarian cysts. The evaluation of CA-125 has a low specificity to diagnose ovarian cancer but the potential to assess, monitor, and evaluate the responses of ovarian cancer therapy [7]. The production of IL6 is a result of proinflammatory cytokines, such as TNFα. In ovarian cancer, IL6 has a direct stimulus towards cancer cells via various mechanisms that contribute to the cell cycle and cancer cell growth [8]. Human epididymis protein 4 (HE4) is a whey acid protein precursor for epididymis secretory E4 with a molecular weight of 11 kDa. HE4 is expressed in various normal tissues, including respiratory and reproduction tissues. HE4 is overexpressed in ovarian epithelial cancer cells. Studies reported good performances of circulating HE4 for ovarian cancer detection and showed a promising role as a prognostic biomarker. In vitro and in vivo studies reported that HE4 plays a role in several molecular pathways associated with cell proliferation, tumor growth, and metastasis in ovarian cancer. Furthermore, the combination of CA-125 and HE4 had a high accuracy in detecting ovarian cancer and disease progression [9].

The main aim of this study is to investigate CA-125 and HE4 levels as the ovarian cancer markers and IL6 as a pro-inflammatory factor that contributes to the cell cycle and growth of ovarian cancer cells to predict tumor resectability in pre-operative patients with advanced epithelial ovarian cancer.

## Materials and methods

This cross-sectional study was conducted in the polyclinic, oncology and gynecology inpatient room of Dr. M. Jamil Padang Hospital from June until December 2022. The inclusion criteria were patients admitted to polyclinic, oncology and gynecology inpatient room, Dr. M. Jamil Padang Hospital with the advanced stage of epithelial ovarian cancer based on pathological anatomy results and willing to participate in this study. The exclusion criteria were (1) patients admitted to polyclinic, oncology and gynecology inpatient room, Dr. M. Jamil Padang Hospital with the advanced stage of epithelial ovarian cancer, but lost to follow-up; (2) accompanied with other malignant; (3) received pre-operative chemotherapy or radiotherapy; (4) diagnosed with renal and heart disease, stroke, diabetes mellitus, hyperthyroidism, hypothyroidism, and other treated or undergoing therapy of metabolic diseases; (5) and patients with benign tumor based on pathological anatomy result.

This study involved patients’ medical reports, and all medical matters relating to this research are confidential. The ethical implications of this study followed the provisions of the Declaration of Helsinki and were approved by the ethical committee of Dr. M. Jamil Padang Hospital (Approval number: 774/UN.162.KEP-FK/2021). The participants approved and signed the informed consent.

### Sample analysis

The advanced epithelial ovarian cancer stage was measured under a microscope based on histology results FIGO stages IIIB–IVA [10]. Post-operative tumor resectability of optimal resection ≤ 1 cm and suboptimal resection > 1 cm [11]. IL6, CA-125, and HE4 were measured using ECLIA (electrochemiluminescence immunoassay).

### Statistical analysis

All data were collected and analyzed using a computer program SPSS (Statistical Package for the Social Sciences). In the descriptive analysis, categorical data were reported in frequency distribution and percentage. Numerical data of pre-operative IL6, CA-125, and HE4 were presented as mean and standard deviation. Univariate and bivariate analyses were performed. Saphiro Wilks test was used to test data normality. Categorical data were assessed using Chi-square and Mann-Whitney tests. Numerical variable correlations were analyzed using Pearson Correlation tests. While the correlation between numerical and nominal variables was analyzed using the Eta correlation test. A p-value of <0,05 was considered a significant correlation. The cut-off value of serum IL6, CA-125, and HE4 was determined with a ROC curve. Sensitivity was defined as the number of patients with suboptimal resections correctly identified divided by the total number of patients with suboptimal resections. Specificity was defined as the number of patients with optimal resection correctly identified divided by the total number of patients with optimal resection.

## Results

### Patients’ characteristics

Table 1 shows the patients’ characteristics. The participants were dominated by age 40–60 years old (57,8%) with a BMI of 18,5–25 kg/m2 (51,6%) and without a history of menopause (51,3%). According to the parity, most of the patients did not have a pregnancy history (37,5%).

**Table 1:** Patients’ characteristics.

Variable	n (64)	%
**Age (years)**		
<19	1	1,6
19–39	14	21,9
40–64	37	57,8
>64	12	18,8
**BMI (kg/m** ^**2**^ **)**		
<17	1	1,6
17-<18.5	7	10,9
18.5–25	33	51,6
>25–27	10	15,6
>27	13	20,3
**Parity**		
0	24	37,5
<3	17	26,6
3–5	16	25
>5	7	10,9
**Menopause**		
Yes	30	46,9
No	34	53,1
**Resectability**		
<1 (optimal)	38	59,4
> 1 (suboptimal)	26	40,6

### Lab profiles

Table 2 shows lab profiles of hemoglobin, erythrocyte, leukocyte, thrombocyte, APTT, albumin, protein, IL6, CA-125, and HE4.

**Table 2:** Lab profiles.

Variable	Mean	SD	Median	Min-max	95% CI	Normality
Hemoglobin (g/dL)	11,25	1,70	11,2	7,20–15,10	10,82–11,67	0,20
Erythrocyte (10^12^/L)	4,26	0,65	4,26	2,52–5,72	4,09–4,42	0,20
Leukocyte (10^9^/L)	8,70	3,47	7,96	2,72–19,13	7,84–9,57	0,00
Thrombocyte (10^9^/L)	375,031	120,13	362	135–875	345,03–405,04	0,06
APTT (seconds)	26,56	8,07	26,80	2,30–80,50	24,54–28,57	0,00
Albumin (mg/dL)	3,95	0,64	4,1	1,80–5,50	3,79–4,11	0,07
Protein (g/L)	7,30	1.30	7,50	0,40–9,60	6,98–7,63	0,00
IL6 (pg/ml)	158,41	99,44	99,44	55,05–444,56	133,57–183,24	0,00
CA-125 (U/ml)	16406,73	11412,21	12252,50	6148–60768	13556,05–19257,42	0,00
HE4 (pmol/L)	473,14	398,50	321,58	145,95–2016,58	373,59–572,68	0,00

### Tumor marker profiles

Table 3 shows the differences between IL-6, CA-125, and HE4 based on tumor resectability. There was a significant difference in IL-6 levels between patients with tumor resectability of suboptimal resection > 1 cm vs optimal resection < 1 cm (1328 vs 752 pg/ml; p<0,001). There was a significant difference in CA-125 levels between patients with tumor resectability of suboptimal resection > 1 cm vs optimal resection < 1 cm (1260,5 vs 819,5 U/ml; p<0,001). There was a significant difference in HE4 levels between patients with tumor resectability of suboptimal resection > 1 cm vs optimal resection < 1 cm (1320 vs 760 pmol/L; p<0,001).

**Table 3:** The profiles of IL-6, CA-125, and HE4 levels based on tumor resectability.

Variable	Resectability		p-value
	<1 cm (optimal)	>1cm (suboptimal)	
IL6 (pg/ml)	752	1328	0,000
CA-125 (U/ml)	819,5	1260,5	0,000
HE4 (pmol/L)	760	1320	0,000

The correlation between tumor markers (IL6, CA-125, HE4) with tumor resectability is shown in Table 4. There was a positive correlation between IL-6 (r = 0,832; p-value = 0,000), CA-125 (r = 0,716; p-value = 0,000), or HE4 (r = 0,716; p-value = 0,000) with tumor resectability.

**Table 4:** Correlation between IL-6, CA-125, or HE4 with tumor resectability.

Variable	Coefficient correlation R	p-value
IL6 (pg/ml)	0,832	0,000
CA-125 (U/ml)	0,716	0,000
HE4 (pmol/L)	0,716	0,000

Table 5 shows the ROC analysis of IL-6, CA-125, HE4, ROMA, and ROMA + IL-6. Both markers of ROMA and ROMA + IL6 had lower sensitivity (61,50%) and specificity (60,50%) compared to IL-6 (92,3% and 92,15%), CA-125 (88,55% and 89,5%), and HE4 (96,2% and 97,4%).

**Table 5:** ROC analysis using IL-6, CA-125, HE4, ROMA, and ROMA + IL-6.

Marker	AUC	P-value	95%CI	Sensitivity	Specificity	COP
IL-6	0,989	0,00	0,971–1	92,3%	92,15%	128,53 pg/ml
CA-125	0,921	0,00	0,842–0,999	88,55%	89,5%	13249,50 u/ML
HE4	98,1	0,00	0,946–1	96,2%	97,4%	410,49 pmol/L
ROMA	0,77	0,00	0,647–0,889	61,50%	60,50%	6075.7450
ROMA + IL6	0,77	0,00	0,647–0,889	61,50%	60,50%	6267.8800

### Tumor marker profiles and the outcomes

**Fig 1:**
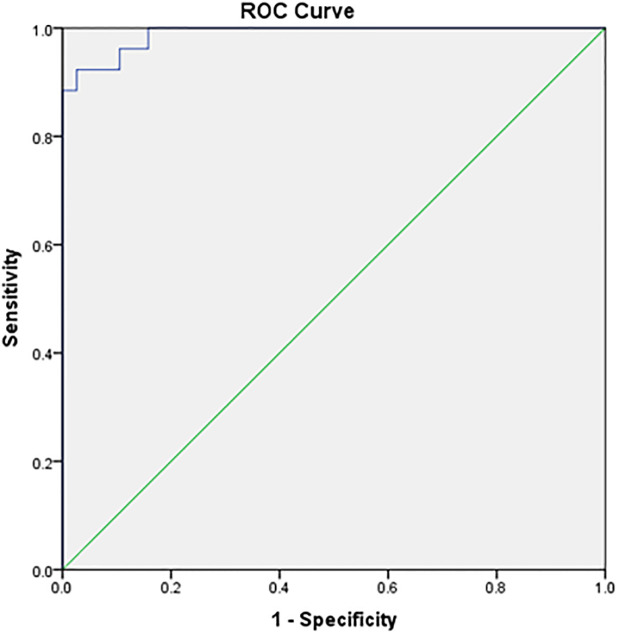
Surgical outcomes of IL6 levels.

Fig 1 displays the ROC and area under a curve of 98,9% with a p-value of <0,001 to predict the surgical outcomes of the patients. The cut-off points of IL6 were 128,53 pg/ml with sensitivity and specificity of 92,3% and 92,1%, respectively.

**Fig 2:**
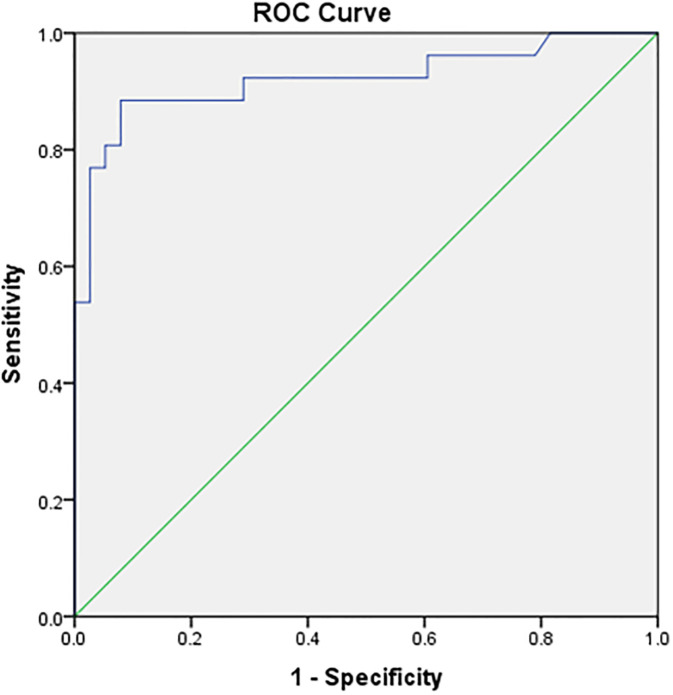
Surgical outcomes of CA-125 levels.

Fig 2 displays the ROC and area under a curve of 92,1% with a p-value of <0,001 to predict the surgical outcomes of the patients. The cut-off points of CA-125 were 13249,50 U/ml with sensitivity and specificity of 88,5% and 89,5%, respectively.

**Fig 3:**
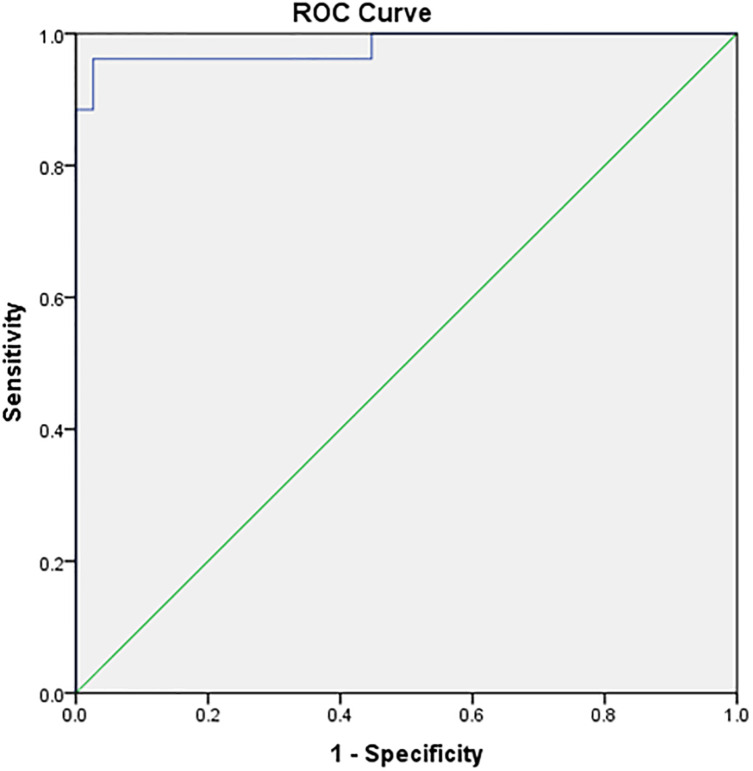
Surgical outcomes of HE4 levels.

Fig 3 displays the ROC and area under a curve of 98,1% with a p-value of <0,001 to predict the surgical outcomes of the patients. The cut-off points of HE4 were 410,40 pmol/L with sensitivity and specificity of 96,2% and 97,4%, respectively.

## Discussion

This study highlights the importance of evaluating tumor marker (IL6, CA-125, and HE4) levels to predict tumor resectability in pre-operative patients with advanced epithelial ovarian cancer. Our study reports that IL6, CA-125, and HE4 levels were higher in the group with tumor resectability of suboptimal > 1 cm. Similar studies have also reported combining tumor markers to predict surgical outcomes in patients with advanced epithelial ovarian cancer. A study by Kampan et al. reported that IL6, CA-125, and HE4 had a high accuracy in diagnosing or predicting the prognosis of optimum cytoreduction in advanced epithelial ovarian cancer with sensitivity and specificity of 100% [8]. Evaluation of IL6 and CA-125 has also been reported to increase the prediction accuracy and determine better management for ovarian cancer [12]. A similar study by Kurniadi et al. found a correlation between pre-operative IL6 and CA-125 levels to predict tumor outcomes [13]. A combination between CA-125 and HE4 has also been reported in a study by Feng et al., who found that the levels of pre-operative CA-125 ≥ 313,6 U/mL and HE4 ≥ 777,1 pmol/L increased the occurrence of suboptimum cytoreduction in the advanced ovarian cancer [14].

According to the Eta correlation test, the difference levels of IL6, CA-125, and HE4 between groups with tumor resectability of suboptimal > 1 cm and optimal < 1 cm also showed a strong positive correlation to the tumor resectability. Additionally, the high sensitivity and specificity of IL6, CA-125, and HE4 showed that evaluation of their levels could be utilized to predict surgical outcomes. Previous studies have also reported high sensitivity and specificity of using a single tumor marker IL6, CA-125, HE4, or combinations to predict ovarian cancer surgical outcomes [8, 13]. The cut-off point value of IL6, CA-125, and CA-125 in this study was also higher than in previous studies. The number of subjects in our study was higher than the other studies may explain the reason for higher sensitivity and sensitivity, as well as the result of the cut-off point value obtained.

The role of IL6 in promoting cellular proliferation in various types of cancer, such as breast, lung, and colorectal cancer, has been well reported. Therefore, the higher IL6 serum levels may also explained the association of pro-inflammatory mediator in the development of advanced ovarian epithelial cancer [15]. The production of cytokines during the inflammation process contributes to the binding and activation of toll-like receptors (TLRs) on the cellular surface. This mechanism involves mitogen-activated protein kinases (MAPKs) p38 and C-jun N-terminal Kineas (JNK) and the transcription factors NF-kB. The MAPK pathway will regulate cellular proliferation, differentiation, growth, migration, and apoptosis by upregulating AP-1, c-Jun, and FOS transcription factors and activation of NF-kB and STATS. Furthermore, NF-kB NF-kB and AP-1 will regulate the production of pro-inflammatory IL6 [16].

Inteleukin-6 is a pro-inflammatory cytokine located in the tumor microenvironment. The production results from local pro-inflammatory cytokine responses, such as TNF-α, expressed mainly in ovarian cancer. IL6 recruits the neutrophils and plays a role in cellular migration, growth, activation, and T- and B-lymphocyte differentiation into plasma cells [17]. Physiologically, IL6 plays an important role in ovarian follicle development in the angiogenesis process by inducing the phosphorylation of STAT3 and MAPK within endothelial cells to enhance cellular migration [18]. Ovarian cancer cells secrete IL6 and IL6Rα (soluble form sIL-6R) through trans-signaling sIL6R-IL6 in the pro-inflammatory microenvironment cells that do not express transmembrane receptor to facilitate angiogenesis via increasing the VEGF expressions [19]. The rapid responses of IL6 to internal and external stimuli make it sensitive to be utilized in the clinical setting [15]. Elevation of IL6 levels may result from ovarian cancer cell proliferation via the activation MAPK-ERK-Akt (protein kinase B) signaling pathway. In the ascites, activation of ERK showed an increase in tumor cellular migration. Additionally, the production of IL6 by M2 macrophage can induce cancer cellular proliferation via STAT3 activation in ascites patients with advanced ovarian cancer [20]. Furthermore, producing MMPs in ovarian cancer cells by IL6 can enhance invasive and tumorigenesis performance [17] The increase of IL6 levels in peritoneum fluid has also shown that the role of JAK-STAT helps the invasive process of ovarian cancer [21].

Although we found the sensitivity and specificity were high in measuring CA-125 to predict surgical outcomes of cytoreduction, some studies also showed that measuring CA-125 was not accurate in predicting optimum cytoreduction surgical outcomes, such as a study by Angioli et al. reported the cut-off point of CA-125 was 414 IU/mL with 58,3% and 84% sensitivity and specificity, respectively [22]. However, a meta-analysis by Kang et al. found that although the levels of CA-125 were not significant to predict optimum cytoreduction, pre-operative CA-125 ≥ 500 IU/mL was considered as the risk factor of suboptimum cytoreduction with 50% of cases required more extensive abdominal surgical procedure. Therefore, a higher pre-operative CA-125 represents the difficulty and complexity of surgical procedures that need to be considered [23].

The increase of CA-125 levels was also reported in other malignancies, such as pancreatic, lung, breast, and colorectal cancer, as well as in ovarian cysts. It was reported that patients with a consistently high CA-125 show the possibility of tumor resectability [17]. An in vitro study found that CA-125 was a potent NK cell inhibitor that reduces CD16 expression on the NK cells (FcγRIII) surface. The binding of CA-125 into the NK cells via galectin-1 facilitated the attachment into the NK cells, promoted metastases, and prevented immune responses [8]. CA-125 also binds with mesothelin, a protein expressed by ovarian, lung, and pancreas cancers, in the normal mesothelium. The mesothelin and protein CA-125 interaction plays an important role in the implantation of ovarian cancer cells into the peritoneum wall [24, 25].

The role of CA-125 in predicting suboptimum cytoreduction has been investigated in a multicenter prospective study involving patients with stages III and IV who underwent primary cytoreduction surgery. Six criteria were classified that associated with suboptimum cytoreduction (tumor resectability > 1 cm), including the levels of CA-125 > 500 IU/mL [26]. Additionally, the use of CA-125 is also potentiated to predict recurrent ovarian cancer.

Early screening is a prevention strategy to reduce the mortality of ovarian cancer. A high expression of CA-125 is often detected earlier than other clinical signs of physical examination and imaging in 80% of ovarian cancer. Currently, the evaluation of CA-125 is involved in the monitoring standard procedures of the patients who underwent remission to detect possible recurrent cancer and to anticipate better treatment strategy [27].

The role of HE4 in predicting the surgical outcomes of ovarian cancer with high sensitivity and specificity has also been reported in previous studies. A study using the combination of tumor markers by Holcomb et al. showed that HE4 was more accurate than other tumor markers, such as CA-125, in predicting epithelial ovarian cancer (88,9% sensitivity and 91,8% specificity) [28]. Similar results have also been reported by Plotti et al. with 75,53% sensitivity and 100% specificity [29]. Additionally, the role of HE4 in monitoring ovarian cancer was more accurate than the benign (98,6% sensitivity and 73,3% specificity) [30].

Previous studies reported the necessity to evaluate early HE4 levels to predict optimum cytoreduction of advanced epithelial ovarian cancer. A prospective study by Angioli et al. in 57 advanced ovarian epithelial cancer showed that HE < 262 pmol/L had 86,1% sensitivity and 89,5% specificity with positive and negative predictive values of 93,9% and 77%, respectively, to predict the success of optimum cytoreductive surgery. Furthermore, HE4 < 262 pmol/L and ascites volume < 500mL had 100% sensitivity and 89,5% specificity with positive and negative predictive values of 94% and 100%, respectively [22]. A similar study was also reported by Shen et al., which also showed that the detection of HE4 was even better than CA-125 in predicting the resectability of ovarian cancer [31]. A Study by Moore et al. reported that HE4 levels could be utilized as a single tumor marker with sensitivity and specificity of 72,9% and 95%, respectively [32]. Furthermore, other studies also reported the same results showing that single HE4 levels detection was more accurate than the combination with other tumor markers (especially CA-125) [28, 30].

The human epididymis protein 4 (HE4) gene is expressed with an exceeded amount in ovarian cancer. The elevation of HE4 was reported in almost all ovarian cancer patients (31/32 patients with > 150 pmol/L) [33]. Therefore, HE4 is a promising tumor marker to diagnose, evaluate the prognosis, and follow up the ovarian cancer patients. The immunoreactivity of HE4 showed the highest levels in various normal human tissues, including the epithelial glands of the genital tract [34]. Various in vitro and in vivo studies have been conducted to investigate the role of HE4 in the proliferation and development of ovarian cancer cells. Wang et al. reported that the given cells with HE4 recombinant group showed significant statistical results regarding the number of colonization among the control group. Additionally, the ablation of HE4 via shRNA will result in the reduction of cellular proliferation. An in vitro study showed the role of HE4 in cellular proliferation by regulating the cell cycle, particularly the transition of G0/G1. The loss of HE4 will terminate G0/G1 cell cycle and the G1 to S transition phase. Conversely, the stimulus of HE4 recombinant will enhance the number of cells in the G2/M phase [35].

In the management to predict tumor resectability, the evaluation of tumor marker levels (IL-6, CA-125, and HE4) is paramount importance, particularly to achieve sub-optimal debulking surgery [36]. However, in patients with low probability of achieving optimal debulking surgery, neoadjuvant chemotherapy followed by internal debulking surgery increases the chance of optimal surgery [37]. As debulking surgery is the primary outcome of most patients with advanced ovarian cancer, different approaches have been developed to improve outcomes for ovarian cancer patients, including the incorporation of targeted therapy, such as anti-VEGF agents, immunotherapy, and PARP inhibitors [38]. For instance, PARP inhibitors have emerged as a significant breakthrough in both newly diagnosed and recurrent cases, especially in patients with BRCA mutations [39]. Furthermore, the success of immunotherapy in various cancer types has sparked interest in its potential application in platinum-resistant ovarian cancer [40]. These novel therapies offer hope for patients facing limited treatment options and hold the potential to improve long-term outcomes.

The limitation in this study that we conducted in a single center with 63 patients involved. A multicenter study with larger samples is required for the future studies.

## Conclusion

Measuring IL6 (cut-off points = 128,53 pg/ml; 92,3% sensitivity and 92,1% specificity), CA-125 (cut-off points = 13249,50 U/ml; 88,5% sensitivity and 89,5% specificity), and HE4 (cut-off points = 410,40 pmol/L; 96,2% sensitivity and 97,4% specificity) levels is useful for clinician to predict tumor resectability in the preoperative patients with advanced epithelial ovarian cancer.

## Supporting information

S1 Checklist STROBE statement—Checklist of items that should be included in reports of observational studies. (DOCX)

S1 File (PDF)

S2 File (PDF)

## References

[1] Torre LA, Trabert B, DeSantis CE, Miller KD, Samimi G, Runowicz CD, et al. Ovarian cancer statistics, 2018. CA Cancer J Clin. 2018;68(4):284–96. doi: 10.3322/caac.21456 29809280 PMC6621554

[2] Huang J, Chan WC, Ngai CH, Lok V, Zhang L, Lucero-Prisno DE 3rd, et al. Worldwide Burden, Risk Factors, and Temporal Trends of Ovarian Cancer: A Global Study. Cancers (Basel). 2022;14(9). doi: 10.3390/cancers14092230 35565359 PMC9102475

[3] Winarto H, Welladatika A, Habiburrahman M, Purwoto G, Kusuma F, Utami TW, et al. Overall Survival and Related Factors of Advanced-stage Epithelial Ovarian Cancer Patients Underwent Debulking Surgery in Jakarta, Indonesia: A Single-center Experience. Open Access Macedonian Journal of Medical Sciences. 2022;10(B):265–80.

[4] Dochez V, Caillon H, Vaucel E, Dimet J, Winer N, Ducarme G. Biomarkers and algorithms for diagnosis of ovarian cancer: CA125, HE4, RMI and ROMA, a review. Journal of Ovarian Research. 2019;12(1):28. doi: 10.1186/s13048-019-0503-7 30917847 PMC6436208

[5] Sölétormos G, Duffy MJ, Othman Abu Hassan S, Verheijen RH, Tholander B, Bast RC Jr., et al. Clinical Use of Cancer Biomarkers in Epithelial Ovarian Cancer: Updated Guidelines From the European Group on Tumor Markers. Int J Gynecol Cancer. 2016;26(1):43–51. doi: 10.1097/IGC.0000000000000586 26588231 PMC4679342

[6] Llueca A, Serra A, Rivadulla I, Gomez L, Escrig J. Prediction of suboptimal cytoreductive surgery in patients with advanced ovarian cancer based on preoperative and intraoperative determination of the peritoneal carcinomatosis index. World J Surg Oncol. 2018;16(1):37. doi: 10.1186/s12957-018-1339-0 29471831 PMC5824576

[7] Charkhchi P, Cybulski C, Gronwald J, Wong FO, Narod SA, Akbari MR. CA125 and Ovarian Cancer: A Comprehensive Review. Cancers (Basel). 2020;12(12). doi: 10.3390/cancers12123730 33322519 PMC7763876

[8] Kampan NC, Madondo MT, Reynolds J, Hallo J, McNally OM, Jobling TW, et al. Pre-operative sera interleukin-6 in the diagnosis of high-grade serous ovarian cancer. Sci Rep. 2020;10(1):2213. doi: 10.1038/s41598-020-59009-z 32042020 PMC7010756

[9] Furrer D, Grégoire J, Turcotte S, Plante M, Bachvarov D, Trudel D, et al. Performance of preoperative plasma tumor markers HE4 and CA125 in predicting ovarian cancer mortality in women with epithelial ovarian cancer. PLOS ONE. 2019;14(6):e0218621. doi: 10.1371/journal.pone.0218621 31220149 PMC6586345

[10] Berek JS, Kehoe ST, Kumar L, Friedlander M. Cancer of the ovary, fallopian tube, and peritoneum. Int J Gynaecol Obstet. 2018;143 Suppl 2:59–78. doi: 10.1002/ijgo.12614 30306591

[11] Song YJ. Prediction of optimal debulking surgery in ovarian cancer. Gland Surg. 2021;10(3):1173–81. doi: 10.21037/gs-2019-ursoc-08 33842263 PMC8033079

[12] Amer H, Kartikasari AER, Plebanski M. Elevated Interleukin-6 Levels in the Circulation and Peritoneal Fluid of Patients with Ovarian Cancer as a Potential Diagnostic Biomarker: A Systematic Review and Meta-Analysis. J Pers Med. 2021;11(12). doi: 10.3390/jpm11121335 34945807 PMC8704427

[13] Kurniadi AL, Hidayat YM, Suardi D, Susanto H, G N.A.W, Prayitno HSH. Peran Serum IL-6 dan CA-125 Prabedah sebagai Prediktor Resektabilitas Tumor pada Kanker Ovarium Tipe Epitel. Indonesian Journal of Cancer. 2018;11:151–8.

[14] Feng LY, Liao SB, Li L. Preoperative serum levels of HE4 and CA125 predict primary optimal cytoreduction in advanced epithelial ovarian cancer: a preliminary model study. J Ovarian Res. 2020;13(1):17. doi: 10.1186/s13048-020-0614-1 32050995 PMC7014747

[15] Kampan NC, Xiang SD, McNally OM, Stephens AN, Quinn MA, Plebanski M. Immunotherapeutic Interleukin-6 or Interleukin-6 Receptor Blockade in Cancer: Challenges and Opportunities. Curr Med Chem. 2018;25(36):4785–806. doi: 10.2174/0929867324666170712160621 28707587

[16] Hu X, li J, Fu M, Zhao X, Wang W. The JAK/STAT signaling pathway: from bench to clinic. Signal Transduction and Targeted Therapy. 2021;6(1):402. doi: 10.1038/s41392-021-00791-1 34824210 PMC8617206

[17] Browning L, Patel MR, Horvath EB, Tawara K, Jorcyk CL. IL-6 and ovarian cancer: inflammatory cytokines in promotion of metastasis. Cancer Manag Res. 2018;10:6685–93. doi: 10.2147/CMAR.S179189 30584363 PMC6287645

[18] Rašková M, Lacina L, Kejík Z, Venhauerová A, Skaličková M, Kolář M, et al. The Role of IL-6 in Cancer Cell Invasiveness and Metastasis—Overview and Therapeutic Opportunities. Cells [Internet]. 2022; 11(22). 10.3390/cells11223698 PMC9688109 36429126

[19] Yang X, Huang M, Zhang Q, Chen J, Li J, Han Q, et al. Metformin antagonizes ovarian cancer cells malignancy through MSLN mediated IL-6/STAT3 signaling. Cell Transplantation. 2021;30:09636897211027819. doi: 10.1177/09636897211027819 34238029 PMC8274104

[20] An Y, Yang Q. Tumor-associated macrophage-targeted therapeutics in ovarian cancer. International Journal of Cancer. 2021;149(1):21–30. doi: 10.1002/ijc.33408 33231290

[21] Savant SS, Sriramkumar S, O’Hagan HM. The Role of Inflammation and Inflammatory Mediators in the Development, Progression, Metastasis, and Chemoresistance of Epithelial Ovarian Cancer. Cancers (Basel). 2018;10(8). doi: 10.3390/cancers10080251 30061485 PMC6116184

[22] Angioli R, Plotti F, Capriglione S, Aloisi A, Montera R, Luvero D, et al. Can the preoperative HE4 level predict optimal cytoreduction in patients with advanced ovarian carcinoma? Gynecol Oncol. 2013;128(3):579–83. doi: 10.1016/j.ygyno.2012.11.040 23220563

[23] Kang S, Kim TJ, Nam BH, Seo SS, Kim BG, Bae DS, et al. Preoperative serum CA-125 levels and risk of suboptimal cytoreduction in ovarian cancer: a meta-analysis. J Surg Oncol. 2010;101(1):13–7. doi: 10.1002/jso.21398 20025071

[24] Hilliard TS. The impact of mesothelin in the ovarian cancer tumor microenvironment. Cancers. 2018;10(9):277. doi: 10.3390/cancers10090277 30134520 PMC6162689

[25] Kakimoto S, Miyamoto M, Einama T, Matsuura H, Iwahashi H, Ishibashi H, et al. Co-expression of mesothelin and CA125 is associated with the poor prognosis of endometrial serous carcinoma and mixed carcinomas including serous carcinoma. Pathology & Oncology Research. 2020;26(4):2299–306. doi: 10.1007/s12253-020-00823-1 32468249

[26] Merlo S, Besic N, Drmota E, Kovacevic N. Preoperative serum CA-125 level as a predictor for the extent of cytoreduction in patients with advanced stage epithelial ovarian cancer. Radiology and Oncology. 2021;55(3):341–6. doi: 10.2478/raon-2021-0013 33675192 PMC8366730

[27] Putri K, Tambunan B, Sandhika W. Diagnostic value of CA-125 in patients with epithelial ovarian cancer at the DR. Soetomo General Hospital Surabaya in 2016. Indonesian Journal of Clinical Pathology and Medical Laboratory. 2019;25:145.

[28] Holcomb K, Vucetic Z, Miller MC, Knapp RC. Human epididymis protein 4 offers superior specificity in the differentiation of benign and malignant adnexal masses in premenopausal women. Am J Obstet Gynecol. 2011;205(4):358.e1–6. doi: 10.1016/j.ajog.2011.05.017 21722869

[29] Plotti F, Capriglione S, Terranova C, Montera R, Aloisi A, Damiani P, et al. Does HE4 have a role as biomarker in the recurrence of ovarian cancer? Tumour Biol. 2012;33(6):2117–23. doi: 10.1007/s13277-012-0471-7 22875782

[30] Chen X, Zhou H, Chen R, He J, Wang Y, Huang L, et al. Development of a multimarker assay for differential diagnosis of benign and malignant pelvic masses. Clin Chim Acta. 2015;440:57–63. doi: 10.1016/j.cca.2014.11.013 25447698

[31] Shen Y, Li L. Serum HE4 superior to CA125 in predicting poorer surgical outcome of epithelial ovarian cancer. Tumour Biol. 2016;37(11):14765–72. doi: 10.1007/s13277-016-5335-0 27629144

[32] Moore RG, McMeekin DS, Brown AK, DiSilvestro P, Miller MC, Allard WJ, et al. A novel multiple marker bioassay utilizing HE4 and CA125 for the prediction of ovarian cancer in patients with a pelvic mass. Gynecol Oncol. 2009;112(1):40–6. doi: 10.1016/j.ygyno.2008.08.031 18851871 PMC3594094

[33] Chan JK, Tian C, Fleming GF, Monk BJ, Herzog TJ, Kapp DS, et al. The potential benefit of 6 vs. 3 cycles of chemotherapy in subsets of women with early-stage high-risk epithelial ovarian cancer: an exploratory analysis of a Gynecologic Oncology Group study. Gynecol Oncol. 2010;116(3):301–6. doi: 10.1016/j.ygyno.2009.10.073 19945740

[34] Liu J, Han L, Sun Q, Li Y, Niyazi M. Meta-analysis of the diagnostic accuracy of HE4 for endometrial carcinoma. European Journal of Obstetrics & Gynecology and Reproductive Biology. 2020;252:404–11. doi: 10.1016/j.ejogrb.2020.07.015 32711295

[35] Wang H, Zhu L, Gao J, Hu Z, Lin B. Promotive role of recombinant HE4 protein in proliferation and carboplatin resistance in ovarian cancer cells. Oncol Rep. 2015;33(1):403–12. doi: 10.3892/or.2014.3549 25354091

[36] Arab M, Jamdar F, Sadat Hosseini M, Ghodssi- Ghasemabadi R, Farzaneh F, Ashrafganjoei T. Model for Prediction of Optimal Debulking of Epithelial Ovarian Cancer. Asian Pac J Cancer Prev. 2018;19(5):1319–24. doi: 10.22034/APJCP.2018.19.5.1319 29802693 PMC6031811

[37] Tangjitgamol S, Manusirivithaya S, Laopaiboon M, Lumbiganon P, Bryant A. Interval debulking surgery for advanced epithelial ovarian cancer. Cochrane Database Syst Rev. 2016;2016(1):Cd006014. doi: 10.1002/14651858.CD006014.pub7 26747297 PMC8602973

[38] Perez-Fidalgo JA, Grau F, Fariñas L, Oaknin A. Systemic treatment of newly diagnosed advanced epithelial ovarian cancer: From chemotherapy to precision medicine. Critical Reviews in Oncology/Hematology. 2021;158:103209. doi: 10.1016/j.critrevonc.2020.103209 33388455

[39] Giannini A, Di Dio C, Di Donato V, D’Oria O, Salerno MG, Capalbo G, et al. PARP Inhibitors in Newly Diagnosed and Recurrent Ovarian Cancer. Am J Clin Oncol. 2023. doi: 10.1097/COC.0000000000001024 37314974

[40] Bogani G, Lopez S, Mantiero M, Ducceschi M, Bosio S, Ruisi S, et al. Immunotherapy for platinum-resistant ovarian cancer. Gynecol Oncol. 2020;158(2):484–8. doi: 10.1016/j.ygyno.2020.05.681 32518015

