# Supplementary material for: The Role of Interleukin 6 (IL6), Cancer Antigen—125 (CA-125), and Human Epididymis Protein 4 (HE4) to predict tumor resectability in the advanced epithelial ovarian cancer patients
Source: PLoS One. 2023 Oct 4;18(10):e0292282. doi: 10.1371/journal.pone.0292282 (PMC10550129; doi:10.1371/journal.pone.0292282)
Supplement: S1 File — (PDF) [file pone.0292282.s002.pdf]

KEMENTERIAN PENDIDIKAN, KEBUDAYAAN,
RISET DAN TEKNOLOGI
UNIVERSITAS ANDALAS
FAKULTAS KEDOKTERAN
KOMISI ETIK PENELITIAN
Alamat : Kampus Universitas Andalas, Limau Manis Padang Kode Pos 25163
Telepon : 0751-31746, Faksimile. : 0751-32838, Dekan : 0751-39844
Laman ; http://fk.unand.ac.id e-mail : dekanat@fk.Unand.ac.id

KETERANGAN LOLOS KAJI ETIK
DESCRIPTION OF ETHICAL APPROVAL

No : 774/UN.16.2/KEP-FK/2022

Tim Komisi Etik Penelitian Fakultas Kedokteran Universitas Andalas, dalam upaya melindungi Hak Azazi dan Kesejahteraan Subjek Penelitian kedokteran/kesehatan, telah mengkaji dengan teliti protokol penelitian dengan judul : *The Research Ethics Committee of Medical Faculty Andalas University, in order to protect human rights and welfare of medical/health research subject, has carefully reviewed the research protocol entitled :*

Prediksi Resektabilitas Kanker Ovarium Tipe Epitel Stadium Lanjut dengan Pemeriksaan Kadar IL-6, CA-125 dan HE4

Nama Peneliti Utama : dr. Reyhan Julio Azwan
Principal Researcher

Nama Institusi : Program Studi Dokter Spesialis Obstetri dan Ginekologi
Institution

Protokol Penelitian tersebut dapat disetujui pelaksanaannya.
and approved the research protocol.

Padang, 13 Juni 2022

Dekan Fakultas Kedokteran Universitas Andalas
Dean of Medical Faculty Andalas University

Dr. dr. Afriwardi, SH. Sp.KO, MA
NIP 196704211997021001

Ketua
Chairman

Dr. dr. Yuliarni Syafrita, SpS (K)
NIP 196407081991032001

Keterangan/*notes:*
Keterangan lolos kaji etik ini berlaku satu tahun dari tanggal persetujuan.
This ethical approval is effective for one year from the due date.
Jika ada kejadian serius yang tidak diinginkan (KTD) harus segera dilaporkan ke Komisi Etik Penelitian.
If there are Serious Adverse Events (SAE) should be immediately reported to the Research Ethics Committee.
