# Supplementary material for: The Role of Interleukin 6 (IL6), Cancer Antigen—125 (CA-125), and Human Epididymis Protein 4 (HE4) to predict tumor resectability in the advanced epithelial ovarian cancer patients
Source: PLoS One. 2023 Oct 4;18(10):e0292282. doi: 10.1371/journal.pone.0292282 (PMC10550129; doi:10.1371/journal.pone.0292282)
Supplement: S2 File — (PDF) [file pone.0292282.s003.pdf]

**DESCRIPTION OF ETHICAL APPROVAL**

**Number: 774/UN.16.2/KEP-FK/2022**

The Research Ethics Committee of Medical Faculty Andalas University, in order to protect human rights and welfare of medical/health research subject, has carefully reviewed the research protocol entitled:

**Prediction of the Advanced Epithelial Ovarian Cancer Resectability by Evaluating IL-6, CA-125, and HE4 Levels**

Principal Researcher : Reyhan Julio Azwan, MD
Institution : Obstetric and Gynecology Medical Specialist Study Program

and approved the research protocol.

Padang, June 13$^{th}$, 2022

Dean of Medical Faculty Andalas University

**Afriwardi, MD, PhD**

Chairman

**Yuliarni Syafrita, MD, PhD**

Notes:
This ethical approval is effective for one year from the due date.
If there are Serious Adverse Events (SAE) should be immediately reported to the Research Ethics Committee
